# Concurrent OX40 and CD30 Ligand Blockade Abrogates the CD4-Driven Autoimmunity Associated with CTLA4 and PD1 Blockade while Preserving Excellent Anti-CD8 Tumor Immunity

**DOI:** 10.4049/jimmunol.1700088

**Published:** 2017-06-23

**Authors:** Maher G. Nawaf, Maria H. Ulvmar, David R. Withers, Fiona M. McConnell, Fabrina M. Gaspal, Gwilym J. Webb, Nick D. Jones, Hideo Yagita, James P. Allison, Peter J. L. Lane

**Affiliations:** * Institute of Immunology and Immunotherapy, University of Birmingham, Birmingham B15 2TT, United Kingdom;; † Institute for Biomedical Research, College of Medical and Dental Sciences, University of Birmingham, Birmingham B15 2TT, United Kingdom;; ‡ Department of Immunology, Genetics and Pathology, Rudbeck Laboratory, Uppsala University, 751 85 Uppsala, Sweden;; § Department of Immunology, Juntendo University School of Medicine, Tokyo 113-8421, Japan; and; ¶ Department of Immunology, MD Anderson Cancer Center, University of Texas, Houston, TX 77030

## Abstract

Although strategies that block FOXP3-dependent regulatory T cell function (CTLA4 blockade) and the inhibitory receptor PD1 have shown great promise in promoting antitumor immune responses in humans, their widespread implementation for cancer immunotherapy has been hampered by significant off-target autoimmune side effects that can be lethal. Our work has shown that absence of OX40 and CD30 costimulatory signals prevents CD4 T cell–driven autoimmunity in Foxp3-deficient mice, suggesting a novel way to block these side effects. In this study, we show that excellent antitumor CD8 T cell responses can be achieved in Foxp3^KO^ mice deficient in OX40 and CD30 signals, particularly in the presence of concurrent PD1 blockade. Furthermore, excellent antitumor immune responses can also be achieved using combinations of Abs that block CTLA4, PD1, OX40, and CD30 ligands, without CD4 T cell–driven autoimmunity. By dissociating autoimmune side effects from anticancer immune responses, this potentially shifts this antitumor approach to patients with far less advanced disease.

## Introduction

Many human cancers are characterized by tumor-infiltrating lymphocytes that include CD4 and CD8 T cells, indicating that tumors do evoke an immune response, probably due to the numerous mutant proteins expressed in cancerous cells (1, 2). However, two well-characterized mechanisms exist to protect tumors from attack by the host immune system. Foxp3-dependent regulatory T cells (Tregs), specific for self-antigens expressed in medullary thymic epithelium (3, 4), can dominantly negatively regulate CD4 and CD8 anticancer responses. Second, in addition to Tregs, many tumors also express the inhibitory ligand PDL1, which can directly inhibit tumor-infiltrating PD1-expressing CD8 T cells from killing their tumor targets.

To counter these inhibitory pathways that prevent effective antitumor immunity, therapies have been developed in humans to block Treg function. The main effector pathway by which Tregs work is through expression of CTLA4 (5), and CTLA4 blockade (6) and PD1 blockade (7) have been shown to be efficacious in some patients with melanoma, particularly when used in combination (8, 9), with 61% of patients in the latter study getting a clinically relevant responses. However, these studies also highlighted dose-dependent toxicity associated with autoimmunity and correlated with efficacy. Toxicity led to grade 3 (requiring hospitalization) and grade 4 (life-threatening) side effects in 54% of treated patients and limited the extent to which dosage could be escalated. The lethal CD4-driven autoimmunity seen in CTLA4-deficient mice (10) shows that at best CTLA4 blockade must be incomplete in human cancer immunotherapy, and although the autoimmunity associated with PD1 deficiency is relatively mild (11), the human melanoma studies show that the autoimmunity is augmented by blocking both CTLA4 and PD1 pathways. The mechanism by which CTLA4-blocking Abs works is controversial. Some studies in humans and mice have demonstrated that they deplete intratumoral Tregs via Fc receptor–mediated mechanisms (12–14) rather than only working by blocking CTLA4. Indeed, depletion of Tregs in adult mice is associated with rapid-onset autoimmunity (15), which might explain at least some of the effects for the off-target autoimmunity observed with CTLA4-blocking Abs. The story with adult CTLA4 depletion is contentious, with studies showing mild (16) to quite severe autoimmunity (17) in mice. However, note that in humans, haploinsufficiency of CTLA4 is associated with significant autoimmunity (18, 19), so blockade of CTLA4 could act synergistically with Treg depletion to promote the CD4-driven autoimmunity seen in mice and humans lacking Tregs (20, 21). We have previously shown that the CD4-driven fatal autoimmunity seen in Foxp3-deficient mice is abrogated in the absence of OX40 and CD30, whereas CD8 immunity was preserved. This raised the possibility that blocking OX40 and CD30 signaling could attenuate CD4-driven autoimmunity without compromising CD8 antitumor responses.

## Materials and Methods

### Mice

For tumor experiments, wild-type (C57BL/6, Charles River Laboratories), CD30^KO^ OX40^KO^ (double knockout [dKO], C57BL/6), and CD30^KO^ OX40^KO^ Foxp3^KO^ (triple knockout [tKO], C57BL/6) female mice (6–8 wk old) were bred and maintained in the animal facilities at the Biomedical Services Unit (University of Birmingham, U.K.). The dKO and tKO mice were generated as described previously (22, 23). In experiments where mAbs were injected, female C57BL/6 mice were used for experiments.

### Murine cell culture and preservation

The murine melanoma B16-F10 cell line (24) was obtained from American Type Culture Collection and has not since been tested for mycobacterial contamination. This cell line is not on the International Cell Line Authentication Committee database of commonly misidentified cell lines. It was cultured in T75 vent cap tissue culture flasks (Sarstedt) using 15 ml of 1× DMEM (Life Technologies) supplemented with 10% heat-inactivated FBS (Sigma-Aldrich) and 100 IU/ml Penicillin, 100 μg/ml Streptomycin (Life Technologies). The cells were harvested in log phase of their growth before injection into mice.

### Subcutaneous injection of mice

Female mice aged 6–8 wk were used for the tumor melanoma model. When possible, age-matched littermates were used, and mice were allocated randomly to treatment groups. B16-F10 melanoma cell suspension was prepared as described above. Mice were anesthetized and the right dorsal flank areas were shaved and treated with disinfectant, prior to s.c. injection of 500,000 B16-F10 cells resuspended in 100 μl of PBS into the right dorsal flank. Tumor growth was measured biweekly using calipers. The mouse body weight was monitored and tumor volumes were calculated using the equation *V* = π/6 × *l* × *d*^2^, where *V* indicates volume, *l* refers to the long diameter, and *d* is the short diameter. Mice were sacrificed when the tumor size was >12.5 mm in diameter.

### Preparation of cell suspensions for flow cytometry

Tumors were weighed after excision. Single-cell suspensions were prepared from tumors, spleen, and draining and nondraining inguinal lymph nodes. Tumors and lymph nodes were cut into small pieces and then digested in 1 ml of digestion medium composed of RPMI 1640, 2% FCS, 100 μg/ml DNase (Roche Applied Science), and 1 mg/ml collagenase D (Roche Applied Science) at 37°C for 30 min. Splenocytes were prepared by physical disaggregation with filtering through a strainer followed by lysis in Gey’s solution. To reduce cell clumping, 1 mM/ml EDTA (Sigma-Aldrich) was added to the digested suspension, which was then filtered through a 70-μm cell strainer (Falcon). Cells were then resuspended in 800 μl of RPMI 1640 for tumors and 400 μl for lymph nodes.

### Flow cytometric analysis

Ninety-six–well rounded-bottom plates (Serilin) were seeded with 200 μl of the cell suspensions, centrifuged at 4°C, 1400 rpm for 3 min, and then washed with cold PBS before staining. The washed cells were first surface stained with Live/Dead near-IR dye (Molecular Probes) for 20 min on ice to separate viable and nonviable cells. The following mAbs were used in this study: anti-CD45 (eBioscience), anti-CD3 PE (clone 500A2; eBioscience), anti-CD4 V500 (clone RM4-5; BD Biosciences), anti-CD8 eFluor 450 (clone 53-6.7; eBioscience), anti-CD25 allophycocyanin (clone PC61; eBioscience), anti-CD44 PE-Cy7 (clone IM7; eBioscience), anti-Foxp3 FITC (clone FJK-16s; eBioscience), anti-PD1 PE-Cy7 (clone RMP1-30; BioLegend, U.K.), anti–granzyme B FITC (clone GB11; BioLegend), ant–IFN-γ PE-Cy7 (clone XMG1.2; eBioscience), and anti-CTLA4 PE-Cy7 (clone UC10-4B9; BioLegend).

Intracellular staining for Foxp3 and CTLA4 was performed following surface staining, fixation, and permeabilization of the cells with a Foxp3 fixation/permeabilization kit (eBioscience). Anti-CD3–coated plates plus CD28 were used to investigate IFN-γ production after a total of 4 h of incubation at 37°C using GolgiStop (BD Biosciences) and a Cytofix/Cytoperm Plus (BD Biosciences) kit. The cells were washed twice and resuspended in 200 μl of FACS buffer (0.1% FBS in PBS). Ten microliters of counting beads (10,000/10 μl) (Spherotech) was added to each sample before analysis according to the manufacturer’s protocol. A Beckman Coulter CyAn ADP was used for acquiring the samples, and FlowJo software was used for analysis of the data.

### Blocking and depleting CD8 Abs

CD8 mAb (clone YTS169.4.2) was used to in vivo deplete CD8 T cells (25). The mAbs used for tumor treatment were: anti-CTLA4 (200 μg), clone 9H10 and clone 9H9, courtesy of J. P. Allison (MD Anderson Cancer Center); anti-PD1 (250 μg), clone RMP1-14; anti-CD30L (250 μg), clone RM153; and anti-OX40L (250 μg), clone RM134L, courtesy of Hido Yagita (Juntendo University School of Medicine, Tokyo, Japan); rat IgG (Sigma-Aldrich) (250 μg) was used as a control. The mAbs were prepared in sterile PBS and injected i.p. twice per week after 5 d of B16-F10 injection (unless otherwise stated).

### Liver function tests

A serum alanine aminotransferase (ALT) Beckman Coulter test kit (OSR 6107) and aspartate aminotransferase (AST) were performed on the sera harvested at sacrifice from experimental mice according to the manufacturer’s protocol and analyzed using a Beckman Coulter AU400 analyzer by the Clinical Biochemistry Unit, Women’s Hospital, Edgbaston, Birmingham, U.K.

### Liver histology

Livers were excised and collected in cold RPMI 1640. The liver tissue was fixed in 40% saline formalin (Adams), dehydrated, embedded in wax, sectioned into 5-μm sections, and stained with H&E. The liver sections were imaged by bright-field microscopy on a Leica DM6000 (Germany) microscope, and areas of lymphocyte infiltration were identified and counted at ×10 and ×20 magnifications.

### Circulating serum autoantibody screening and titration

Blood was collected from mice by cardiac puncture according to UK Home Office regulations. The blood samples were allowed to clot naturally and then centrifuged at 13,000 rpm, 4°C for 15 min. Isolated serum was transferred into new tubes and stored at −20°C until use. Frozen sera were thawed at room temperature and diluted 1:20 in 1× PBS. Commercially prepared 10-spot rat tissue slides (NOVA Lite rat liver, kidney, stomach) were removed from their bags and dried at room temperature for 20 min. The slides were blocked with 10% goat serum (Sigma-Aldrich) in 1% BSA/PBS (50 μl per spot) and incubated for 20 min at room temperature in a dark humidified chamber. The diluted mice sera were applied to the 10-spot slides and incubated as previously for 20 min. Slides were then washed in PBS for 5–10 min, excess fluid was wiped from around spots, and 50 μl per spot of goat anti-mouse IgG FITC was added and incubated for 20 min. The slides were washed and antifade reagent was added; they were covered and then stored in the dark at 4°C until imaging. Positive, negative, and second-step controls were also prepared. Tissues were imaged by fluorescence on a Leica DM6000B (Germany) microscope, and data were interpreted using LAS and Fiji software.

### Statistical analysis

From previous experiments we know that when there is no overlap between experimental results in controls and test mice, groups of five mice will achieve a statistical significance of *p* < 0.05 using nonparametric statistics that do not assume a normal distribution. Based on preliminary experiments, a power calculation was made that suggested an 80% power to detect a significance of *p* < 0.05. Results from in vitro and in vivo experiments were expressed as medians, with results from individual mice shown as symbols. No mice were excluded from experimental analysis. For experiments where mice were injected with various combinations of mAbs, the person who analyzed the results was blinded to which Abs had been given to the different groups. We did not assume for any group that they were normally distributed. Therefore, the Mann–Whitney nonparametric test was used to analyze data using GraphPad Prism software, and *p* < 0.05 was considered statistically significant. FACS plots were analyzed using FlowJo v10 (Tree Star).

## Results

### dKO Foxp3^KO^ (tKO) mice resist B16-F10 melanoma tumor growth but have increased infiltrates of PD1-expressing CD8 T cells

**FIGURE 1.**
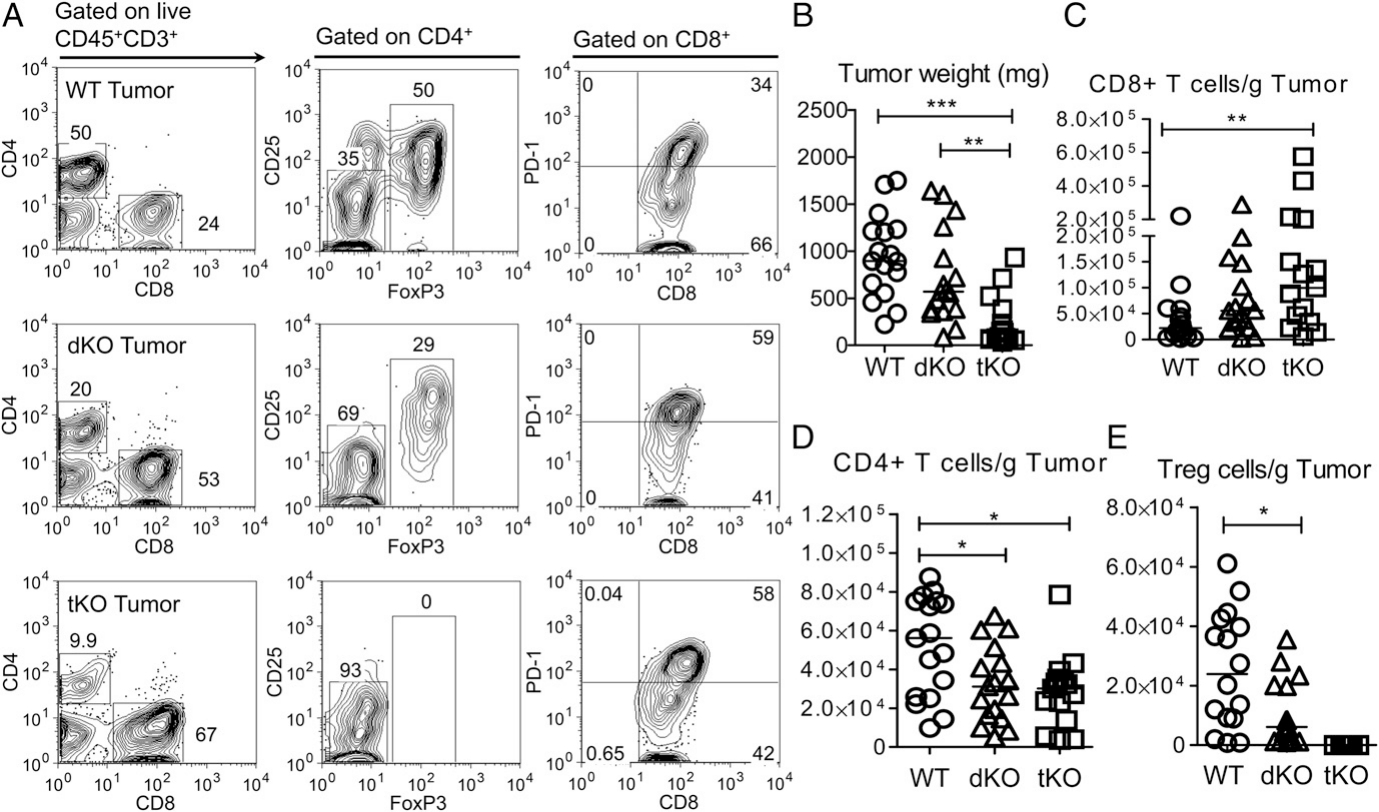
Pattern of tumor growth and cellular infiltrates in WT, dKO, and tKO mice. Tumors were harvested 21 d after injection of 5 × 10^5^ melanoma B16-F10 cells in WT, dKO (OX40^KO^CD30^KO^), and tKO (OX40^KO^CD30^KO^Foxp3^KO^) mice and analyzed. (**A**) Typical flow cytometric analysis for each group of mice, (**B**) tumor weight at harvest, (**C**) CD8 tumor infiltrates, (**D**) CD4 tumor infiltrates, and (**E**) Foxp3^+^ infiltrates are shown. Statistical analysis was performed using a Mann–Whitney nonparametric test. Data are representative of three independent experiments, lines shows medians, and each symbol represents one mouse (*n* = 16, 17, 15) in each group, respectively. *p < 0.05, **p < 0.01, ***p < 0.005.

**FIGURE 2.**
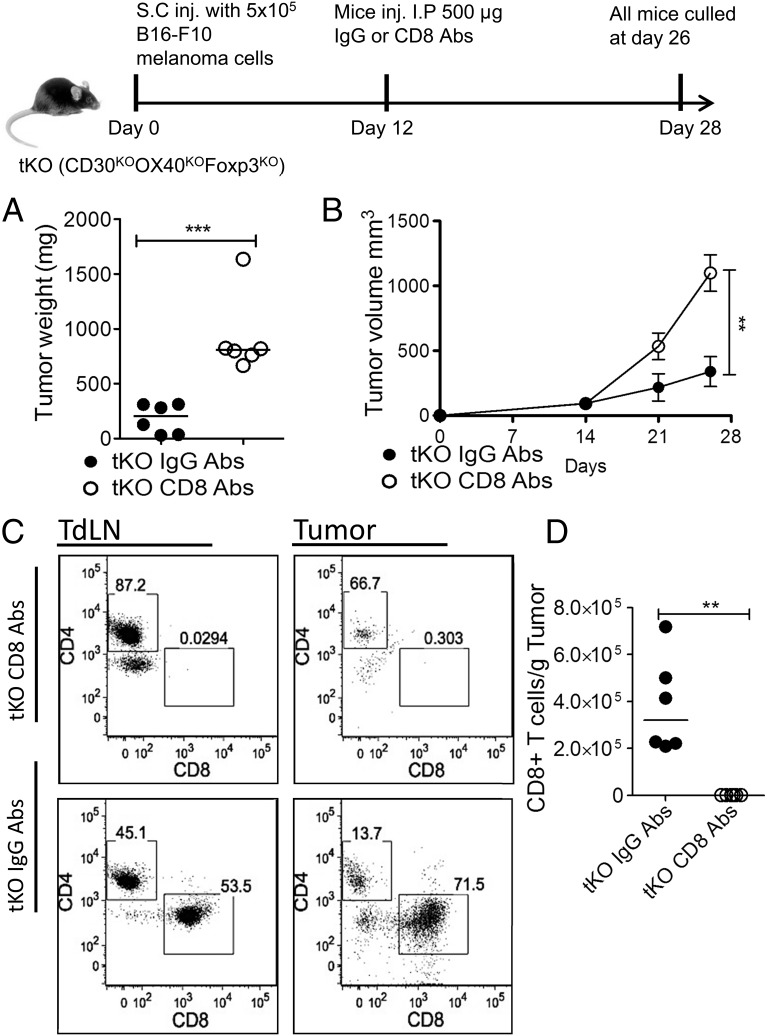
The suppression of tumor growth response in tKO mice is substantially CD8-dependent. B16-F10 cells (5 × 10^5^)were injected s.c. into the right flank of tKO mice. At day 12, mice were injected i.p. with either control or anti-CD8 mAbs. (**A**) Tumor volume at days 14, 21, and 26; line shows means with error bars ± SEM. (**B**) Tumor weight at excision; each data point represents one mouse, and line shows medians. (**C**) FACS analysis of tumor-draining lymph nodes (TdLN) and tumor- infiltrating T cells, gated on CD3^+^ cells. (**D**) CD8 tumor infiltrates. This experiment confirms previous observations by others that the antitumor response is CD8-dependent (27). Data are representative of two independent experiments (*n* = 6, 6) in each group. Statistical analysis was performed using a Mann–Whitney nonparametric test. **p < 0.01, ***p < 0.005.

Tumor growth cannot be assessed in Foxp3^KO^ mice, as they die of lethal CD4-driven autoimmune disease by 4 wk of age. To assess the impact of Treg deficiency, we therefore compared growth of the melanoma line B16-F10 in WT C57BL/6 dKO (OX40 and CD30-deficient) and tKO (dKO and Foxp3^KO^) mice. Mice were inoculated with 5 × 10^5^ melanoma cells in the flank, and tumor growth was monitored using calipers. At day 21, all groups of mice were sacrificed, and tumors were excised. Compared to WT control mice and dKO mice, tKO mice, which lack all Treg function, had significantly reduced tumor growth (dKO, *p* < 0.023; WT, *p* < 0.003) (Fig. 1B, Supplemental Fig. 1). Representative FACS data (Fig. 1A, Supplemental Fig. 2A) and T cell infiltrates per gram of tumor are shown in Fig. 1C–E, and T cell populations in tumor inguinal-draining lymph nodes are shown in Supplemental Fig. 2B–D. The normal CD4/CD8 T cell ratio seen in WT tumors was inverted in tKO and dKO mice (Fig. 1A), supporting the role for OX40 and CD30 signals in CD4 effector function and in agreement with the abrogation of CD4 but not CD8 IFN-γ expression reported previously for CD30^KO^OX40^KO^ mice (23). However, CD8 tumor-infiltrating cells showed significant expression of the inhibitory receptors PD1 and CTLA4 (Fig. 1A, Supplemental Fig. 3A, 3B). Enumeration of T cell subsets per gram of tumor showed a significant increase in CD8 T cells (*p* < 0.009) in tKO versus WT mice, with dKO mice showing intermediately increased infiltrates of CD8 T cells (Fig. 1C). In contrast, there was a statistically nonsignificant trend toward decreases in non-Treg CD4^+^ T cells in dKO and tKO tumors compared with WT (Fig. 1D), as well as in Treg numbers in dKO mice (Fig. 1E). Similar findings have been reported for an OX40-deficient human (26). CD8 T cells infiltrating tKO mice tumors showed similar expression of the activation marker CD44, granzyme B, and IFN-γ compared with WT and dKO (Supplemental Fig. 3C–F). The inhibition of tumor growth in tKO mice was substantially dependent on CD8 T cells, as CD8 depletion in tKO mice 12 d after injection with B16 melanoma cells led to rapid growth of B16 melanoma compared with control-injected mice (Fig. 2), as has been found by others (27, 28).

### PD1 blockade acts synergistically in Foxp3^KO^ dKO (tKO) mice, and with WT mice injected with blocking mAbs to CTLA4, and OX40L and CD30L, to prevent tumor growth

**FIGURE 3.**
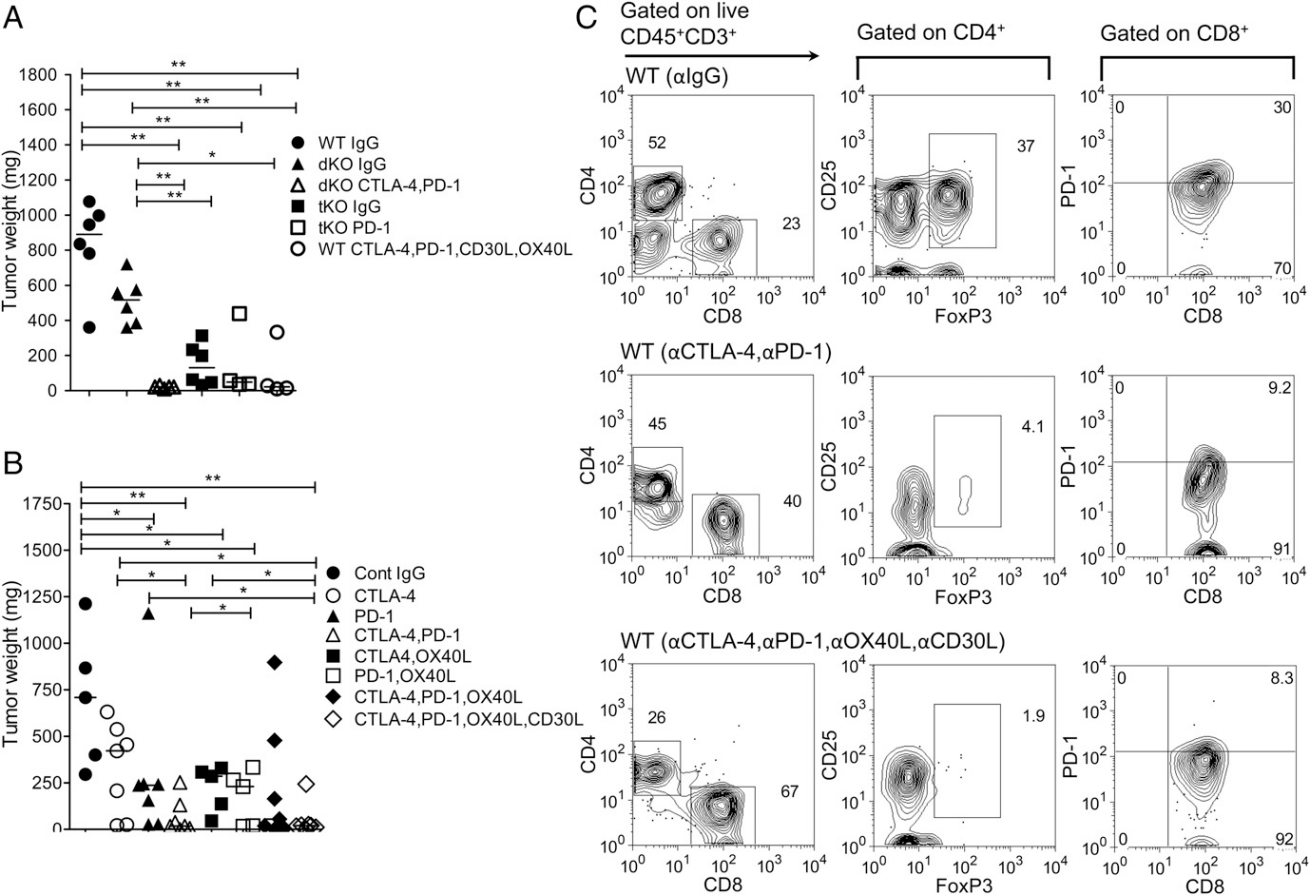
Harvested tumor weights (milligrams) and representative tumor-infiltrating lymphocytes in mice treated with control and blocking Abs. (**A**) Day 22 harvested tumor weights (milligrams) after excision in WT, dKO, and tKO mice injected with blocking mAbs. Mice were injected with B16-F10 as previously. Three days after melanoma inoculation, mice were injected with control or blocking Abs twice a week for 3 wk. The significance was calculated using a Mann–Whitney nonparametric test. Data are representative of three independent experiments; the line shows medians in each group. *p < 0.05, **p < 0.005. (**B**) Harvested tumor weights (milligrams) at day 42 or when tumor size reached 12 mm by caliper measurement (humane endpoint). Five days after melanoma cells were injected, control or blocking mAbs were initiated twice a week until the experiment was terminated at day 42. Each data point represents one mouse; line shows medians. Data are representative of three independent experiments. Statistical analysis was performed using a Mann–Whitney nonparametric test. *p < 0.05, **p < 0.01. (**C**) Representative flow cytometry analysis of harvested WT control tumors, WT mice treated with CTLA4 and PD1, and WT mice treated with CTLA4, PD1, OX40L, and CD30L mAbs.

**FIGURE 4.**
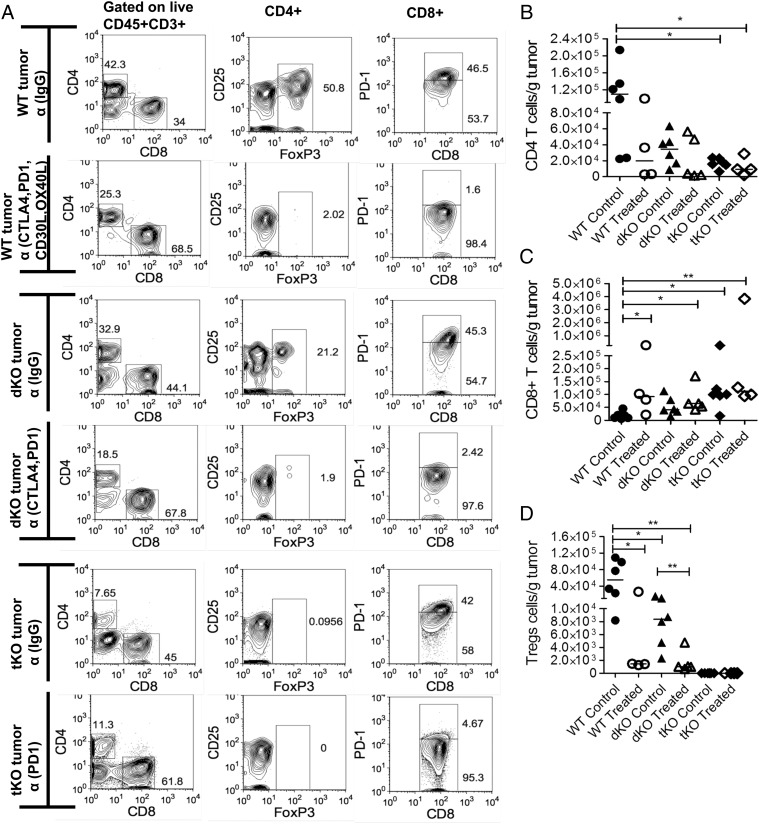
Effect of CTLA4 and PD1 mAbs on tumor-infiltrated T cell populations in WT, dKO, and tKO mice. All tumors were harvested on day 22. (**A**) Typical flow cytometric analysis for each group. (**B**) CD4^+^ tumor infiltrates, (**C**) CD8^+^ tumor infiltrates, and (**D**) Foxp3^+^ tumor infiltrates are shown. Each dot represents one mouse; line shows medians. Statistical analysis was performed using a Mann–Whitney nonparametric test. Data are representative of three independent experiments. *p < 0.05, **p < 0.01.

Because PD1 was expressed on tumor-infiltrating CD8 T cells, we sought to understand whether addition of PD1-blocking mAbs augmented the antitumor effect. WT, dKO, and tKO mice were injected with B16 cells as previously, and after 3 d of injection the mice were treated with mAbs twice per week for 3 wk. Tumor growth was monitored weekly and all mice were sacrificed at day 22. Treatment with combined CTLA4 and PD1 Abs in dKO mice virtually abrogated tumor growth and increased infiltrates of CD8 T cells as well as its effector functions compared with dKO mice treated with control mAbs (*p* < 0.004) (Figs. 3A, 4A, 4C, Supplemental Fig. 3E, 3F).

To tease apart the effect further, a cohort of 6- to 8-wk-old female C57BL/6 mice was inoculated with tumor, and 5 d later, after the tumors had become established, combinations of blocking (CTLA4, PD1, OX40L, CD30L) or control mAbs were administered twice weekly until tumors reached the tumor size limit (12 mm in diameter) or day 42, at which point the remaining animals were sacrificed and analyzed. In mice that received control mAbs, there was rapid growth of tumors (Fig. 3B, Supplemental Fig. 1B). Injection of CTLA4-blocking Abs slowed down tumor growth compared with controls, but they were not as effective as PD1-blocking Abs (*p* < 0.03) or the combination of PD1 and CTLA4 blockade (*p* < 0.003). CTLA4 and OX40L blockade was more efficacious than CTLA4 blockade on its own (*p* < 0.03), and PD1 and OX40L combined was equally efficacious as PD1 on its own. Finally, the addition of OX40L- and CD30L-blocking Abs to the PD1- and CTLA4-blocking combination was equally efficacious as combined CTLA4 and PD1 blockade. Analysis of infiltrates in tumors revealed depletion of Foxp3^+^ Tregs in mice injected with CTLA4- and PD1-blocking mAbs as previously reported (13), with an increased proportion of CD8 T cells (Figs. 3C, 4A, 4D). In mice also injected with OX40L- and CD30L-blocking mAbs, the proportion of CD8 T cells was further increased, consistent with a primary role for OX40 and CD30 signals in CD4 T cell survival (22).

### Blocking Abs to CTLA4 and PD1, in combination with OX40L and CD30L Abs, significantly attenuates concomitant CD4 autoimmunity

**FIGURE 5.**
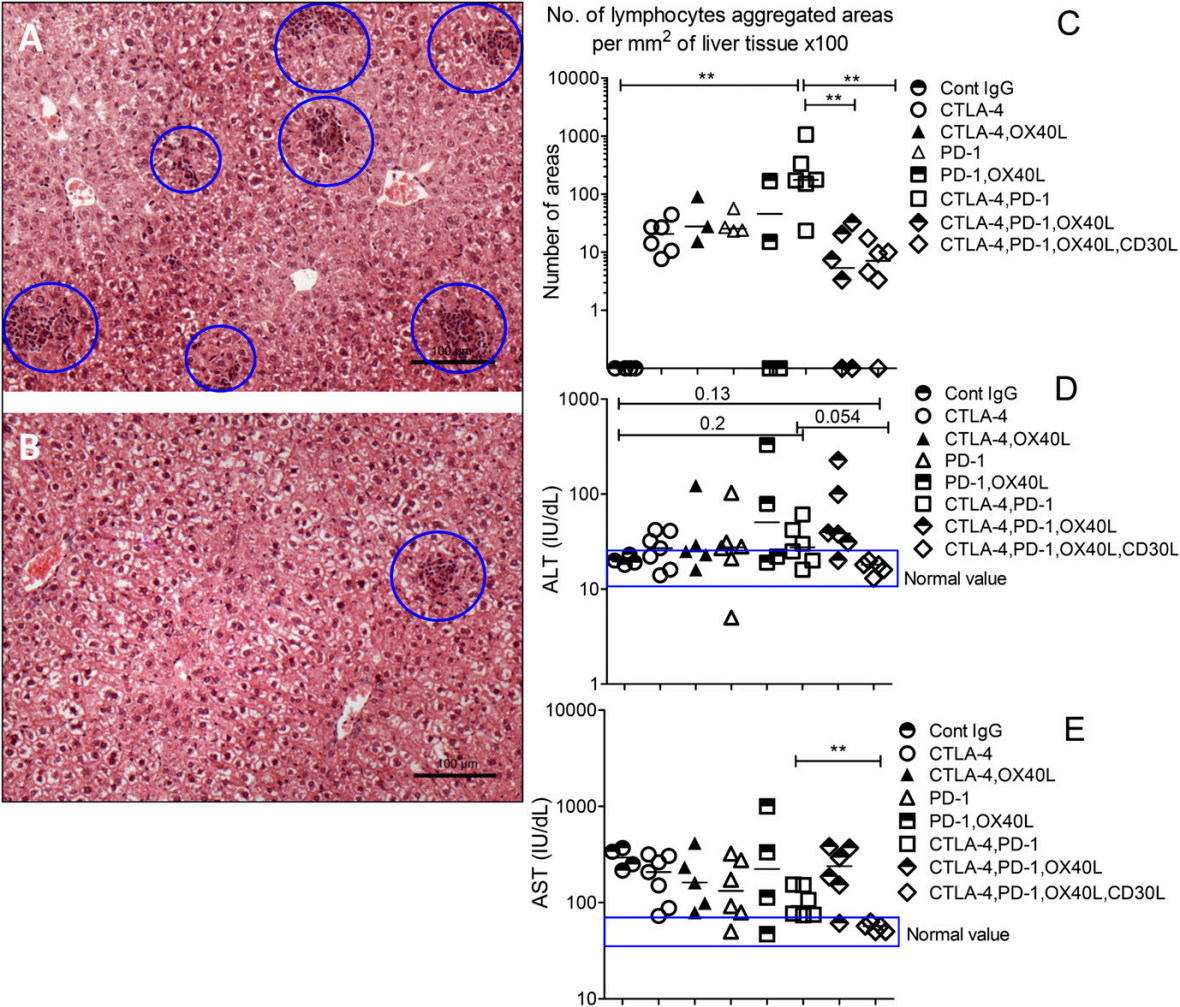
Liver histology and liver function tests after injecting combinations of CTLA4, PD1, OX40L, and CD30L mAbs in tumor-injected C57BL/6 mice. Formalin-fixed liver tissue sections stained with H&E show lymphocyte infiltration associated with immunotherapy using blocking Abs to CTLA4 and PD1 combinations (**A**) and CTLA4, PD1, OX40L, and CD30L combinations (**B**) in B16-F10–injected C57BL/6 mice. (**C**) Quantification of lymphocyte infiltration into B16-F10–transfected WT mice livers treated with blocking Abs. Mice sera values of ALT (**D**) and AST (**E**) were measured as a biomarker of liver cellular injury for C57BL/6 mice. Results are representative of two experiments; each symbol represents one mouse, and line shows medians values. Data represent duplicate tests for the sera (*n* = 4, 7, 5, 6, 4, 6, 6, 6) in each group. Blue rectangles represent normal ranges of ALT and AST serum values. The significance was calculated using a Mann–Whitney nonparametric test. *p < 0.05, **p < 0.01.

The above results indicated that addition of OX40L and CD30L mAbs to combination CTLA4 and PD1 blockade did not interfere with the CD8 antitumor immune response. We have previously shown that OX40L and CD30L mAbs will block the liver immunopathology in Foxp3^KO^ mice (23). To test whether CTLA4 and PD1 blockade caused significant off-target liver immunopathology, we examined lymphocytic infiltrates in explanted livers of mice treated with various combinations of control or blocking mAbs. Compared to control-injected mice, all groups of mice had detectable lymphoid infiltrates, but these were much more extensive in mice that received a combination of CTLA4 and PD1 blockade (*p* < 0.02) (Fig. 5A–C). A significant finding in our studies was that in all mice that received CTLA4 mAbs, intratumoral Tregs were depleted, whereas Tregs in draining lymph node were retained (Figs. 3C, 4A, 4D). This is consistent with previous findings with these CTLA4-blocking Abs (13).

The addition of blocking Abs, either OX40L alone (*p* < 0.01) or in combination with CD30L (*p* < 0.002), reduced liver infiltration to <5% of the level seen with CTLA4 and PD1 blockade, and this correlated with liver function tests, which were normal with OX40L and CD30L treatment and elevated in mice that received just CTLA4 and PD1 (Fig. 5D, 5E). OX40 is the main driver of pathology in Foxp3^KO^ mice (23), and liver infiltrates in mice given CTLA4, PD1, and OX40L were significantly less than in mice treated with CTLA4 and PD1 blockade alone (*p* < 0.008); ALT and AST liver enzymes were raised in this combination, but not when CD30L-blocking mAbs were added, compatible with significant synergy between OX40L and CD30L blockade in ameliorating off-target side effects.

Additionally, we looked at immunofluorescence staining of liver/kidney and stomach for evidence of autoantibodies and saw reduced mean fluorescence intensity in the group of mice that received concurrent OX40L and CD30L blockade in combination with CTLA4 and PD1 mAbs (Supplemental Fig. 4A, 4B)

Final evidence of abrogation of autoimmunity was that vitiligo and white hair at the injection site that were observed in three of seven mice that received CTLA4 and PD1 mAbs (Supplemental Fig. 4C) was not seen with mice that also received OX40L and CD30L blockade (none of seven mice). In a murine model of vitiligo, CD4 T cells have been implicated as the main effector T cells (29).

## Discussion

The above data suggest that a highly effective way to ameliorate the CD4-driven toxicity associated with CTLA4 and PD1 blockade in cancer treatment (8) would be to combine this therapy with blockade of OX40 and CD30 signals, for cancers where CD8 T cells were the main effectors of the anticancer response. In this study, as has been reported elsewhere in mice (12–14) and humans (30), CTLA4 mAb can deplete intratumoral Tregs but not those in the draining lymph node, probably by Fc receptor–dependent mechanisms (12–14), in addition to blocking CTLA4 interactions. Similar results causing intratumoral Treg depletion have been reported with ipilimumab in humans (30). Furthermore, mAbs to OX40 have also been shown to also deplete intratumoral OX40^+^ Tregs (31), suggesting that intratumoral Treg depletion can augment antitumor immune responses. However, there is also evidence for efficacy with CTLA4-blocking Abs of the IgG2 isotype that should not deplete intratumoral Tregs (32), although there is some evidence that IgG2 mAbs can also trigger myeloid but not NK-mediated Ab-dependent cellular cytotoxicity (33).

Although we found depletion of Tregs in our study, we cannot exclude an additional role for CTLA4 blockade on tumor-infiltrating T cells. In both CTLA4-deficient (10) and Foxp3-deficient mice (34), autoimmunity is driven by CD4 effector pathology, and depletion of Tregs in adult mice is associated with rapid onset of autoimmune pathology (15). Although the autoimmunity seen in mice is less severe with adult CTLA4 depletion (16, 17), haploinsufficiency of CTLA4 has been associated with human autoimmunity, so it is likely in humans that CTLA4 blockade also drives significant autoimmunity (18, 19).

Additionally, OX40 is important for Treg function (35) (F.M. Gaspal, D.R. Withers, M.G. Nawaf, F.M. McConnell, and P.J.L. Lane, unpublished observations, particularly in combination with CD30), so in theory OX40L and CD30L ligand mAbs would impair Treg function even more than CTLA4 blockade on its own in situations where CTLA4 mAbs were nondepleting of intratumoral Tregs (5), without compromising the antitumor CD8 response or causing further off-target CD4-driven side effects.

OX40 signals are the main driver of autoimmunity in Foxp3^KO^ mice (23), and one potential worry is that blockade of OX40 signals would compromise CD4 immune function, rendering patients susceptible to infection. In this regard it is reassuring that an OX40-deficient patient did not suffer from infections with extracellular bacterial infections, nor did they fail to control common herpesvirus infections (27). Furthermore, we have data showing that dKO mice vaccinated with the pneumococcal vaccine Prevenar were protected from lethal challenge with live pneumococci (A.M. Mitchell, T.J. Mitchell, and P.J.L. Lane, unpublished observations). This suggests that basic protective immunity would not be compromised by OX40 blockade.

In human studies, there was a strong correlation between the dose of PD1- and CTLA4-blocking Abs and anticancer efficacy, but further escalation of dosage was limited by dose-related toxicity (8). Our results indicate that by blocking CD4-driven side effects, the block to dose escalation would be removed. OX40L-blocking mAbs have been studied in asthma trials (36) and although found to be ineffective in that setting, they were shown to be safe. As far as we are aware, CD30L-blocking mAbs have not been studied in human trials, although strategies to block CD30L have been patented (patent WO2013163377 A1, https://patentscope.wipo.int/search/en/detail.jsf?docId=WO2013163377&recNum=244&docAn=US2013038135&queryString=robotics&maxRec=46977). By dissociating CD4-driven side effects from CD8-mediated antitumor responses, we think combination therapy could transform immunotherapy for cancer by bringing these treatments to cancer patients much earlier in their diagnosis, and even as upfront treatment rather than for late-stage metastatic disease.

## Supplementary Material

Data Supplement
