# Supplementary material for: Concurrent OX40 and CD30 Ligand Blockade Abrogates the CD4-Driven Autoimmunity Associated with CTLA4 and PD1 Blockade while Preserving Excellent Anti-CD8 Tumor Immunity
Source: J Immunol. 2017 Jun 23;199(3):974–81. doi: 10.4049/jimmunol.1700088 (PMC5523579; doi:10.4049/jimmunol.1700088)
Supplement: Data Supplement [file JI_1700088.zip › JI_1700088_Supplemental_Figures_1.pdf]

Supplemental data

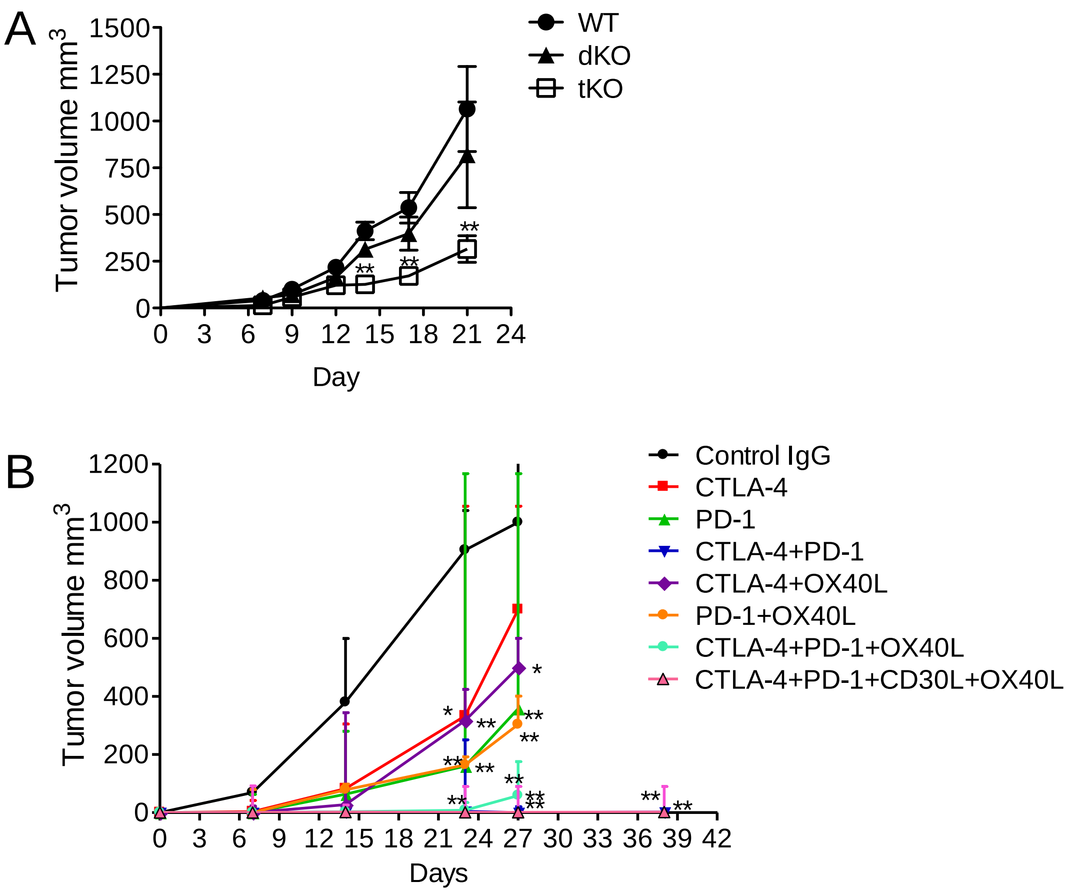

Supplementary figure 1: Kinetics of tumor growth in WT, dKO and tKO mice and in C57BL/6 mice groups treated with blocking mAbs.

(A) WT (C57BL/6), dKO ($CD30^{KO}OX40^{KO}$) and tKO ($CD30^{KO}OX40^{KO}$ $Foxp3^{KO}$) female mice, aged 6-8 weeks, were injected subcutaneously with 5 x 10^5 B16-F10 melanoma cells on the right dorsal flank. Tumours volume was measured at least twice weekly using callipers and the mean of three readings recorded for each mouse in each group. For each group, medians and standard error of means (SEM) are shown, data are representative of three independent experiments.

(B) 5d after tumor injection, combinations of blocking (CTLA4, PD1, OX40L, CD30L) or control mAbs were administered 2x/week weekly until tumor reached 12mm in diameter or on d42. Graph shows medians and error bars and data are representative of three independent experiments.

Statistical analysis was performed using Mann Whitney non parametric test; * ,**,*** indicates significance P<0.05, P<0.01 and P<0.005 respectively.

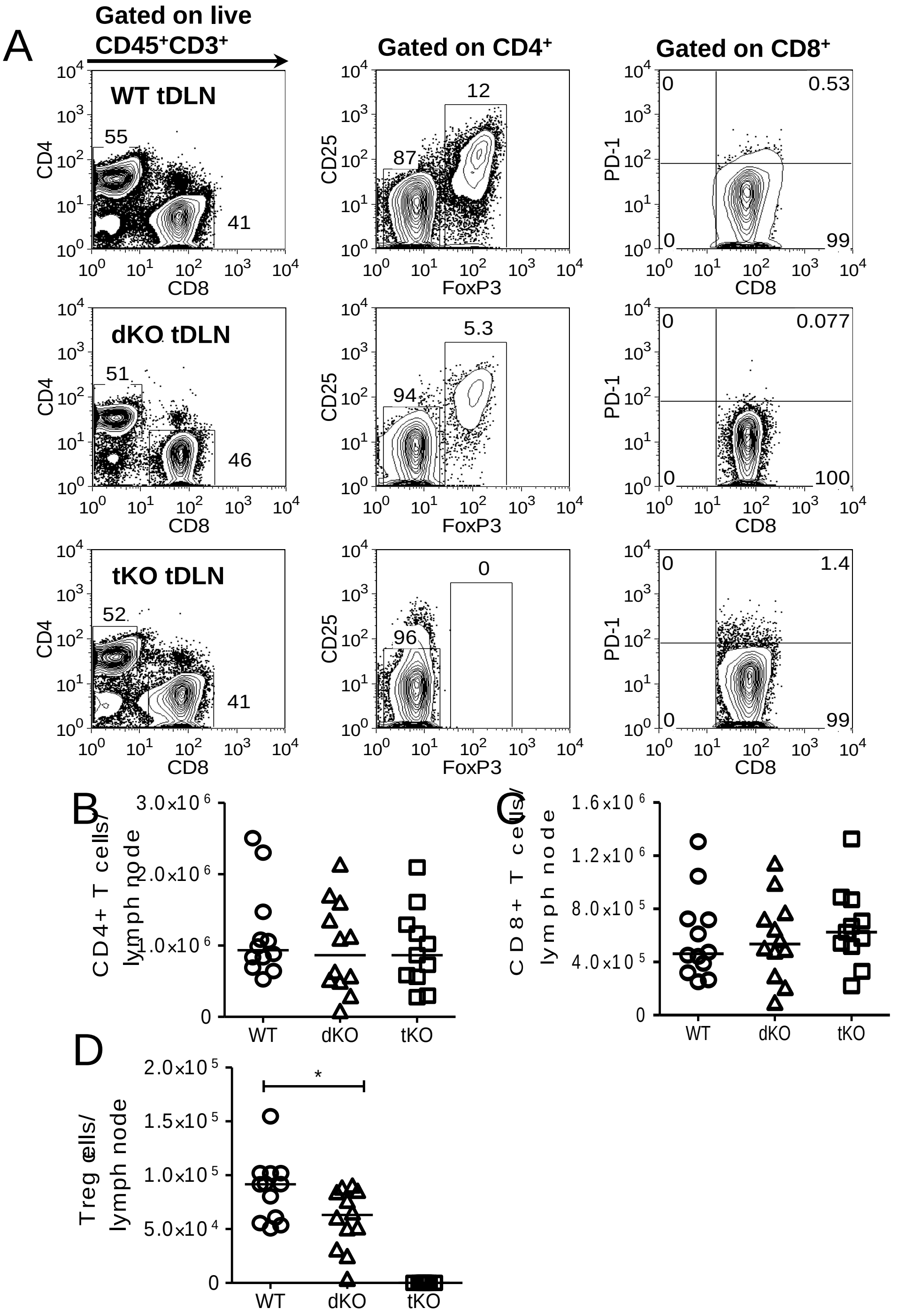

Supplementary figure 2. FACS analysis and T cell numbers of tumor-draining inguinal lymph nodes of of WT, dKO and tKO mice.

Tumors draining inguinal lymph nodes (TdLN) were harvested from the WT (C57BL/6), dKO ($CD30^{KO}OX40^{KO}$) and tKO ($CD30^{KO}OX40^{KO}$ $Foxp3^{KO}$) mice and cells suspension were prepared. Cells were surface stained, fixed and permeabilised and FoxP3 stained. **(A)** Contour plots FACS analysis for tumor draining inguinal lymph nodes (TdLN), (**B**) CD4 count/TdLN, (**C**) CD8 count/TdLN, (**D**) $FoxP3^{+}$ Tregs count/TdLN. Statistical analysis was performed using Mann Whitney non parametric test, * ,**,*** indicates significance P<0.05, P<0.01 and P<0.005 respectively. Data are representative of three independent experiments, n≥7 in each group.

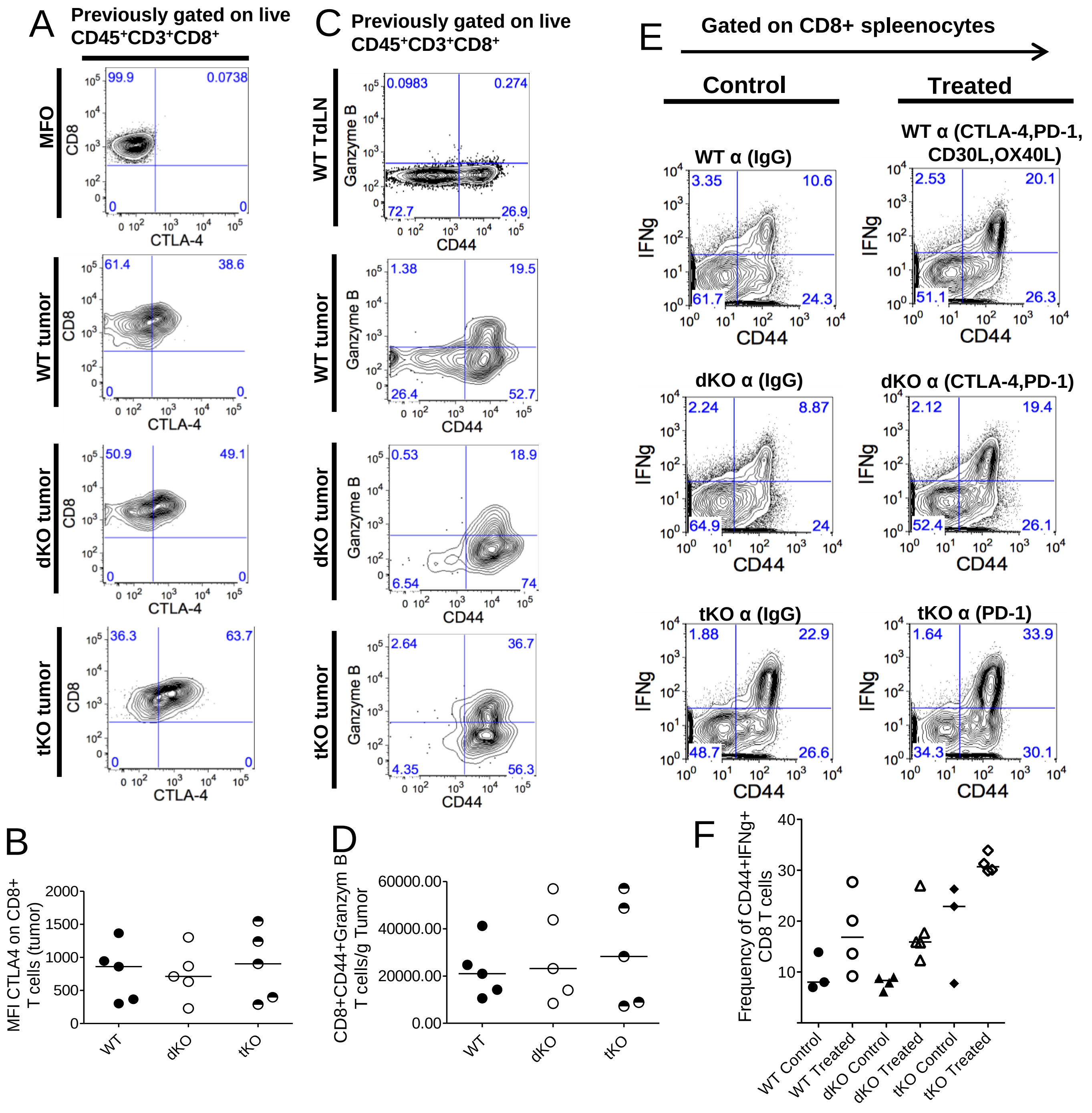

Supplementary figure 3. Intracellular CTLA-4 and Granzyme B expression on tumor infiltrating CD8 T cells in WT, dKO and tKO mice and INF-ɣ production in WT,dKO and tKO mice treated with blocking antibodies.

Tumors were harvested at 22d from WT, dKO and tKO mice. **(A, C)** Typical flow cytometric analysis for each group of mice, **(B)** Mean fluorescent intensity of CTLA4 on CD8 tumor infiltrates, **(D)** CD8+CD44+ Granzyme B+ tumor infiltrates. Data are representative of two independent experiments, lines show medians, each symbol represents one mouse.

Mice treated with monoclonal antibodies anti-(CTLA4, PD1, OX40L, CD30L) or anti-IgG and were sacrificed on day 22. Splenocytes were activated and stained for IFNγ **(E)** Typical flow cytometric analysis for each group of mice, mAbs treated mice (right) and their controls (left), **(F)** Frequency of CD44+IFNγ+ CD8 T cells in spleens. Data are representative of three independent experiments, line shows medians. Statistical analysis was performed using Mann Whitney non parametric test. * ,**,*** indicates significance P<0.05, P<0.01 and P<0.005 respectively.

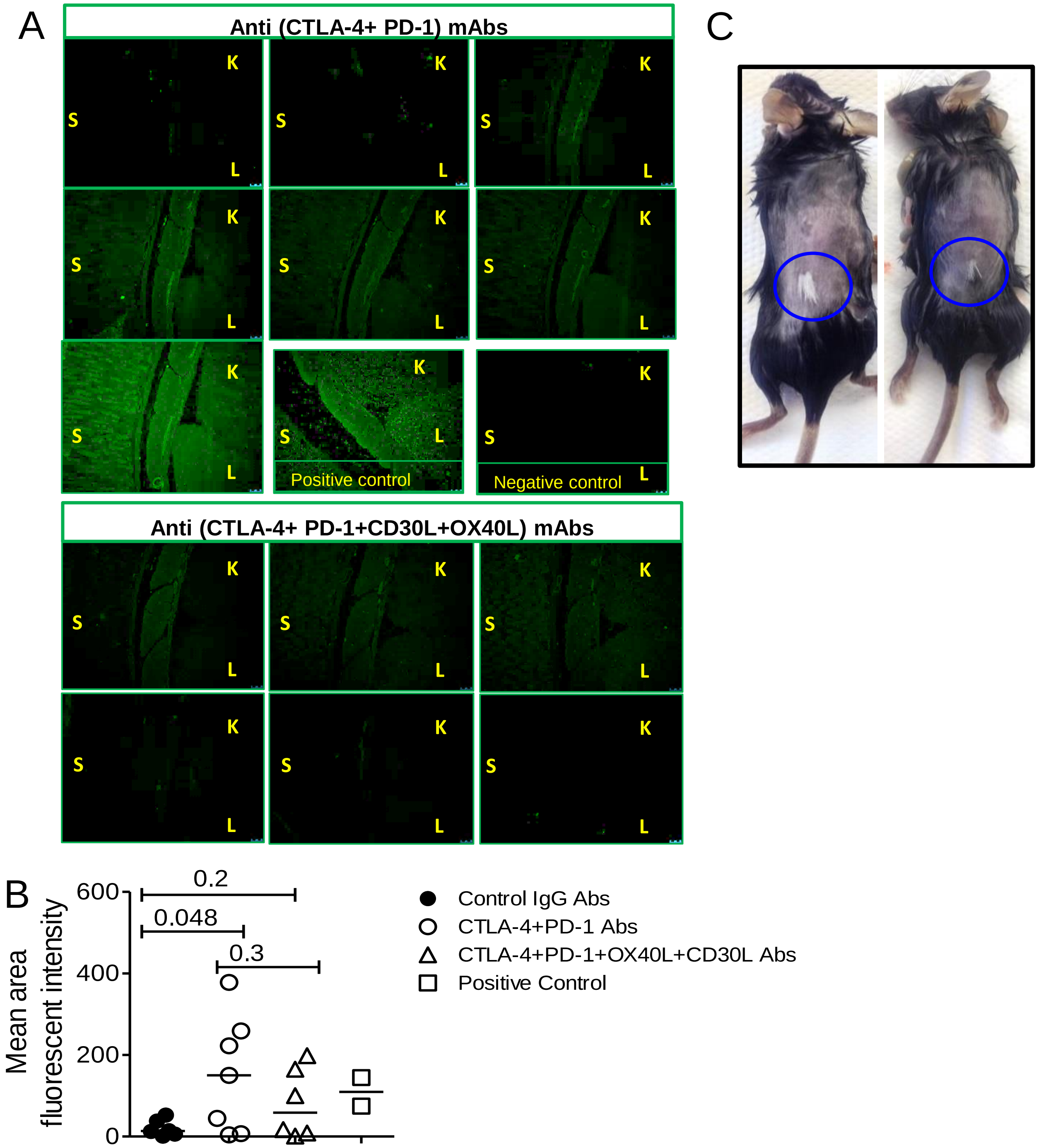

Supplementary figure 4. Vitiligo after injecting combinations of mAbs against CTLA4 and PD1 in tumor injected C57Bl6 mice and immunofluorescent staining on rat tissues sections for CTLA4 and PD1 versus CTLA4, PD1, CD30L, OX40L mAb treated mice.

Mice sera were assessed for autoantibody titters and indirect fluorescence staining on stomach **(S)**, liver **(L)** and kidney **(K)** tissues. Each data point represents one mouse, **(A)** shows fluorescent image for each mouse, **(B)** mean fluorescent intensity per image. **(C)** Representative macrographs of mice injected with (CTLA4, PD1) mAbs combinations shows signs of vitiligo particularly at the site of tumor injection, white hair, blue circles.
Statistical analysis was performed using Mann Whitney non parametric test; * ,**,*** indicates significance $P<0.05$, $P<0.01$ and $P<0.005$ respectively.
